# A Study on the Community and Ecological Characteristics of Benthic Invertebrates in the Ulungu River, Xinjiang, via eDNA Metabarcoding and Morphological Methods

**DOI:** 10.3390/biology14040410

**Published:** 2025-04-12

**Authors:** Qiang Huo, Yuying Ma, Linghui Hu, Qi Liu, Chengxin Wang, Jiaxuan Liu, Daoquan Ren, Zhichao Wang, Baoqiang Wang, Honghui Zeng, Yong Song, Sheng’ao Chen

**Affiliations:** 1 College of Life Sciences and Technology, Tarim University, Alar 843300, China; 10757223082@stumail.taru.edu.cn (Q.H.); 10757223079@stumail.taru.edu.cn (Y.M.); 10757222068@stumail.taru.edu.cn (L.H.); 10757241105@stumail.taru.edu.cn (Q.L.); 10757203067@stumail.taru.edu.cn (C.W.); 10757241109@stumail.taru.edu.cn (J.L.); 119950007@taru.edu.cn (D.R.); wzcdky@126.com (Z.W.); 2 Tarim Research Center of Rare Fishes, Tarim University, Alar 843300, China; 3 Institute of Hydrobiology, Chinese Academy of Sciences, Wuhan 430072, China; wangbq@ihb.ac.cn (B.W.); zhh@ihb.ac.cn (H.Z.)

**Keywords:** Ulungu River, benthic invertebrates, eDNA, morphology, ecological assessment

## Abstract

eDNA metabarcoding has been used for the biomonitoring of benthic invertebrates, but the correct steps to achieve its effectiveness, the stability of the results, and comparisons with morphological methods are still understudied. In this study, morphology and eDNA were studied for benthic invertebrate samples collected at six sites in the Ulungu River Basin. A Mantel test and NMDS analyses were used to test the correlations of the communities obtained via the two methods, the Shannon index was calculated to evaluate the ecological status, and the correlation of the evaluation results was analyzed. The results revealed that eDNA metabarcoding did not detect a greater number of species than the morphological method, that the results from the two methods shared fewer taxa at the family level and below, and that only five taxa were shared at the genus level. The Mantel test and NMDS analyses revealed very significant differences between the communities monitored by the two methods (PERMANOVA, *p* = 0.0056), but eDNA metabarcoding provided richness and abundance data for species that are difficult to identify morphologically, and these findings can be used to supplement the morphological data. The cor.test revealed that there was no significant correlation between the diversity and ecological assessment results of the two methods, and the ecological assessment results of eDNA metabarcoding cannot represent accurate and true ecological conditions. The water sample eDNA-based method and morphological method exhibited low consistency and high complementarity in monitoring benthic invertebrate communities and diversity. More research is still needed on the key links of eDNA sampling, the control of the degradation rate, data utilization, and index development to provide more environmentally friendly and effective monitoring methods for ecological protection, more reliable support for ecological decision-making, and to more adequately respond to the challenges of global environmental change.

## Simple Summary

To investigate the efficiency of eDNA metabarcoding in monitoring benthic macroinvertebrate communities and ecological assessment in river ecosystems, this study compared eDNA metabarcoding results with morphological identification. The findings revealed that eDNA metabarcoding did not detect a greater number of benthic macroinvertebrate taxa compared to traditional methods, and its results did not invariably correspond to the true community structure. However, eDNA metabarcoding demonstrated a higher detection rate for annelids than morphological approaches. Ecological assessments based on eDNA metabarcoding were not always reliable. This study contributes to the advancement and optimization of eDNA-based methods for benthic macroinvertebrate research, enhancing the current knowledge and understanding of eDNA applications in river ecological monitoring, thereby providing stronger support for ecological decision-making.

## 1. Introduction

River ecosystems play a crucial role in sustaining biodiversity, regulating climate patterns, and supporting human societal functions [1,2,3,4]. Anthropogenic stressors on river ecosystems exhibit multi-source composite characteristics: agricultural non-point source pollution disrupts aquatic nitrogen–phosphorus balance, the unauthorized discharge of industrial wastewater leads to an accumulation of heavy metals and persistent organic pollutants, while urban expansion-induced river channel hardening compromises ecological connectivity. Furthermore, wastewater from intensive livestock farming and grazing practices may exacerbate algal blooms and facilitate the dissemination of microbial antibiotic resistance genes, thereby generating compounded ecological risks [5]. The quantitative assessment of threats and impacts on ecosystem integrity and biodiversity necessitates comprehensive evaluations of ecological structure and aquatic ecological status [6]. Ecological indicators refer to natural species that respond to environmental changes and can be used to assess the health of ecosystems [7]. Benthic invertebrates are an important part of aquatic ecosystems and are in the middle of the food chain [8]. They have limited mobility. Their community structure and diversity can reflect the degree of disturbance in the ecosystem and can therefore be used as indicators to quantify ecosystem decline [9,10]. The monitoring of the community structure of benthic invertebrates can be used to assess the ecological status of rivers. In traditional morphological survey methods, much time and a large number of personnel are required for sampling, and it is difficult to capture rare and small organisms [11]. In addition, species identification requires a high level of professionalism. The aquatic insects collected are often in the larval stage. The species identification characteristics below the family classification level are vague, and identification at the genus and species levels is difficult. The accuracy and resolution of the identification results are limited [12]. Therefore, it is necessary to further supplement and improve the survey methods for benthic invertebrates [13,14].

DNA-based molecular methods are powerful monitoring tools [15]. In particular, since the beginning of the 21st century, high-throughput sequencing technology has been developed rapidly and can be used to process many samples at the same time [16]. Compared with traditional Sanger sequencing technology, high-throughput sequencing is faster and less expensive [17]. With the development and application of high-throughput sequencing technology, eDNA metabarcoding has received increasing attention for its potential applications in ecological monitoring and assessment [18,19,20,21,22,23]. Compared with traditional morphological monitoring methods, eDNA metabarcoding can be used to analyze short DNA sequences (usually <500 bp) and classify and assign them via comparison with reference sequence databases to identify species in large samples containing complete organisms or environmental samples (such as water or sediments) [24,25]. Owing to its advantages, such as its noninvasiveness, high sensitivity, efficiency, ease of sampling standardization, and low cost, eDNA metabarcoding has been used to monitor benthic invertebrate diversity and aquatic ecosystems [26].

The Ulungu River is an important inland river in the Junggar Basin in Xinjiang, northwestern China. It originates in Qinghe County in the southern part of the Altai Mountains, flows from east to west through Fuyun County and Fuhai County, and finally flows into Ulungu Lake, the second largest freshwater lake in Xinjiang. The Ulungu River Basin is 821 km long and has a drainage area of 61,400 km^2^ [27]. It is located in a region with a cold, temperate, arid continental climate and relies mainly on mountain rainfall and ice and snow melt for water replenishment [28]. It is the main water source for Fuhai and Qinghe Counties. The Ulungu River and the Irtysh River were originally two independent water systems. However, owing to the “Diversion of the Irtysh River” construction project conducted between 1986 and 1987, a 3 km long canal was dug, making the Ulungu River a tributary of the Irtysh River. The connectivity between different rivers may increase the exchange of biological genes and the genetic diversity of populations, but it may also invade the original ecology, squeeze the original species’ ecological niches, and weaken the heterogeneity of habitats. In recent years, due to the rapid growth of social and economic development, coupled with the low rainfall and high evaporation in the Ulungu River Basin, the surface runoff stability has become poor and water shortages have affected residents and their agricultural irrigation methods near the basin, with frequent dry-ups of the Ulungu River occurring [29]. This has caused the benthic invertebrate communities in the basin to face multiple stresses of habitat heterogeneity and hydrological disturbances, and environmental filtering has led to changes in community structure. The extreme hydrological characteristics and frequent dry-ups of the Ulungu River make morphological operations difficult. The existing morphological-based biological monitoring frequency is difficult to meet the needs of ecological early warning. Therefore, it is urgent to verify the applicability of new monitoring technologies such as eDNA metabarcoding for inland rivers in this arid area.

The aim of this study was to verify the efficiency of the eDNA metabarcoding method in investigating the benthic invertebrates in inland rivers by comparing the consistency and complementarity of eDNA metabarcoding and traditional morphological methods in monitoring benthic invertebrate communities, to study the correlation in assessing ecological status via biological indices, and to provide a reliable new method for river ecosystem surveys to facilitate better water ecological protection. This study expects that eDNA will be able to take advantage of its high sensitivity and monitor species that cannot be detected by the naked eye through morphological observations. Due to limitations such as water flow and degradation, the monitoring results of eDNA are expected to differ from the community structure monitored by morphological observations.

## 2. Materials and Methods

### 2.1. Overview of the Study Area and Field Sampling

**Figure 1:**
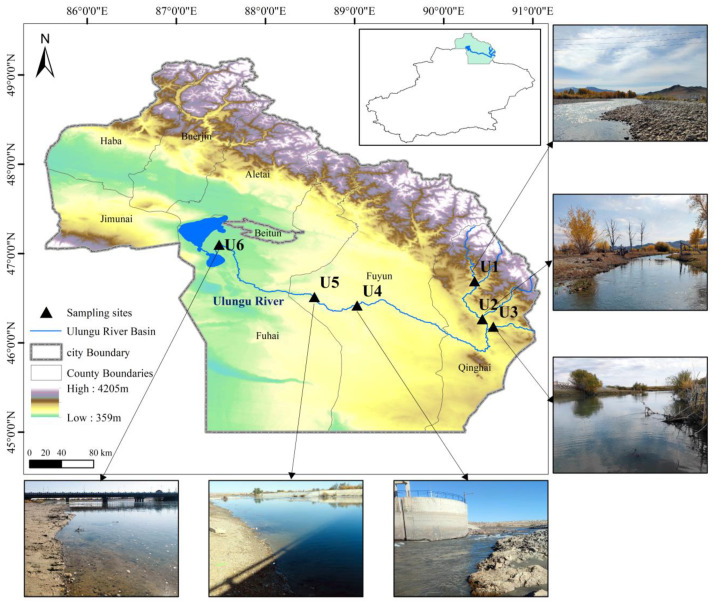
Distribution of eDNA and morphological sample collection sites in the Ulungu River Basin.

In October 2023, based on the previous investigation and the ecological and geographical characteristics of the river, we set up 6 sampling points in the Ulungu River Basin to collect benthic invertebrates. U1, U2, and U3 were set up in different upstream source tributaries. According to the previous investigation, a river dam was built in the river channel of the U4 site. Only by on-site observation can it be found that there is a significant difference in turbidity with other sites and that the sediment content is high. In addition, the U5 and U6 sites were set up in the downstream area of the main stream close to towns with large human interference activities (Figure 1).

The morphological identification method was performed according to the “Chinese Economic Animals” [30] and “Chironomid Larvae of Northern China” [31] documents. A D-shaped net with a size 60 mesh and a 25 cm side length was used to collect morphological samples of benthic invertebrates. Three areas were selected in different habitats for each sampling point, and a total area of 0.25 m^2^ was quantitatively sampled. The selected benthic invertebrates were placed in 50 mL specimen bottles filled with fixative (7% formalin solution). Most benthic invertebrates were identified at the genus level, and a few species were identified at the family or higher taxonomic levels.

eDNA sampling was carried out by collecting three 1 L parallel water samples within the flow range of the river at each site using a water sampler and mixing them. A total of 1 L of the mixed water sample was taken for filtration. The sampling personnel wore sterile gloves and masks throughout the process and used a 0.22 μm filter membrane to obtain water filter membrane samples through a filtration device. The device was disinfected and sterile gloves were replaced after each sample change. The filter membrane samples were stored at −20 °C [32] and brought back to the laboratory for storage at −20 °C prior to molecular processing. The subsequent eDNA extraction and sequencing process was undertaken by Sangon Biotech (Shanghai) Co., Ltd, Shanghai, China. 

### 2.2. Extraction of Total DNA from Membrane Filters

The filter membrane sample was cut into pieces with sterile scissors, lysis buffer was added, and the samples were subsequently disrupted. An appropriate amount of the washed liquid was removed and centrifuged at 12,000 rpm for 2 min. The precipitate was used to extract DNA via the OMEGA (Omega Bio-tek, Inc., Norcross, GA, USA) kit E.Z.N.A^™^ Mag-Bind Soil DNA Kit. Agarose gel electrophoresis was used to detect DNA integrity and a Qubit was used to quantify the DNA sample concentration.

### 2.3. COI Amplification and Sequencing

For this study, a two-round amplification experiment was designed. In the first round of amplification, the genomic DNA was accurately quantified via the Qubit^®^ 4.0 DNA Assay Kit (Thermo Fisher Scientific Inc., Waltham, MA, USA) to determine the amount of DNA to be added to the PCR. The primers used in the PCR were the classic primers mlCOIintF: GGWACWGGWTGAACWGTWTAYCCYCC and dgHCO2198: TAAACTTCAGGGTGACCAAARAAYCA, which were designed on the basis of the mitochondrial COI gene [33,34,35]. In the second round of amplification, Illumina bridge PCR-compatible primers were introduced for amplification. After the PCR products were obtained, library quality control was performed, and the library size was determined via 2% agarose gel electrophoresis. If the electrophoresis bands were clear, the products were deemed to meet the requirements of high-throughput sequencing.

### 2.4. High-Throughput Sequencing Data Analysis

The original sequencing reads, called the raw data, were obtained via high-throughput sequencing. After the primer adapter sequence was removed, the effective data (clean data) of each sample were obtained by filtering and quality control. After the effective data were obtained, the sequences were clustered by OTU. First, nonrepetitive sequences were extracted from the optimized sequence of each sample to reduce the number of redundant calculations in the intermediate analysis process. After the redundant sequences of all samples were merged, the single sequences without duplication were removed. Then, OTU clustering was performed on the nonrepetitive sequences (excluding single sequences) according to 97% similarity, and chimeras were removed during the clustering process to obtain the representative sequence of OTUs. All optimized sequences were aligned to the representative sequence of OTUs, and sequences with a similarity of more than 97% with the representative sequence were selected to finally generate OTU table data.

By using the NCBI NT database, we performed taxonomic annotation and statistical analysis on the representative OTU sequences, used BLASTn v2.10.0 to compare the sequences with the corresponding database, screened out the best alignment results for the sequences, and filtered the alignment results. The sequences with similarity >90% and coverage >90% were selected for subsequent classification, and the sequences that did not meet the conditions were deemed unclassified. Finally, the community composition of each sample was statistically analyzed at each classification level: domain, phylum, class, order, family, and genus.

### 2.5. Construction of the eDNA Database of Local Benthic Invertebrates in the Ulungu River

The MySQL platform was used to initially construct a local eDNA database of benthic invertebrates in the Ulungu River. The database currently contains only the eDNA data obtained in this study. Currently, the database can be connected and shared in a specific way, and it can be further developed and continuously supplemented to serve as an online database that can realize shared queries on the Internet.

### 2.6. Comparison of Community Structure Obtained via eDNA Metabarcoding and Traditional Morphological Methods

The Mantel test is a standard method for testing “matrix correlation” in community ecology. It is suitable for evaluating the global correlation of high-dimensional community matrices, and can process geographic gradient data and avoid spatial autocorrelation interference. The sensitivity of the Bray–Curtis distance index to abundance data can make it more sensitive to capture the compositional differences in community structure (such as species presence/absence and abundance gradient). In order to compare the benthic invertebrate community structure obtained based on traditional morphological and eDNA metabarcoding methods, we calculated the relative abundance data of benthic invertebrates obtained by the two methods. The morphological relative abundance is the ratio of the individual density of the taxonomic unit to the total individual density of the sample, and the eDNA relative abundance is the ratio of the number of OTU reads to the total number of target reads in the sample. The study used the “vegan package” in R v4.4.0 to perform a Mantel test based on the Bray–Curtis distance to test the overall correlation of the communities obtained via the two methods. The Mann–Whitney *U* test was used to analyze the potential differences in taxonomic richness between the two methods at each taxonomic level and the differences in taxonomic richness at each site. Nonmetric multidimensional scaling (NMDS) analysis and permutational multivariate analysis of variance (PERMANOVA) were used to analyze the correlations of benthic invertebrate communities at each site via the vegan package. NMDS can visualize complex species data and then use PERMANOVA to test the significance of differences.

### 2.7. Comparison of EARs Between eDNA Metabarcoding Methods and Traditional Morphological Methods

Ecosystem health can be assessed via a biological index on the basis of the benthic invertebrates. In this study, the Shannon index [36], a diversity index, was used to assess ecosystem health. The Shannon index is calculated as follows:

(1) $$H\prime =-\sum_{n=1}^{n}p_{i}\times \ln p_{i}$$

In the formula, *P_i_* is the proportion of the total sample represented by species i. The evaluation criteria of the Shannon index are divided into four levels: heavy pollution (0 < H’ < 1), moderate pollution (1 < H’ < 2), light pollution (2 < H’ < 3), and clean (H’ < 3). The correlation between the biodiversity obtained via the two methods was tested using the cor.test function in the vegan package in R v4.4.0 [37].

## 3. Results

### 3.1. Consistency or Complementarity of the Two Methods for Monitoring Benthic Invertebrate Communities

After eDNA metabarcoding, 412,985 raw reads were obtained from the six sites in the Ulungu River Basin. After quality control, 384,864 clean reads were obtained. After annotation, 3122 OTUs (including 323,194 clean reads) were obtained. After comparison and allocation with the NCBI nt database, the OTU annotations of nonfreshwater benthic invertebrates were screened, and a total of 54 freshwater benthic invertebrate OTUs belonging to 6 phyla, 10 classes, 16 orders, 27 families, 43 genera, and 48 species were obtained. Using traditional morphological methods, members of 3 phyla, 6 classes, 13 orders, 33 families, and 53 genera were detected; the only phyla detected were Arthropoda, Annelida, and Mollusca. By combining the two methods, 38 genera were detected using eDNA metabarcoding alone, 48 genera were detected using traditional morphological methods alone, and 5 genera were detected by both methods, namely, *Micronecta*, *Microtendipes*, *Cricotopus*, *Limnodrilus*, and *Radix*, belonging to 3 phyla, 3 classes, 4 orders, and 4 families.

**Figure 2:**
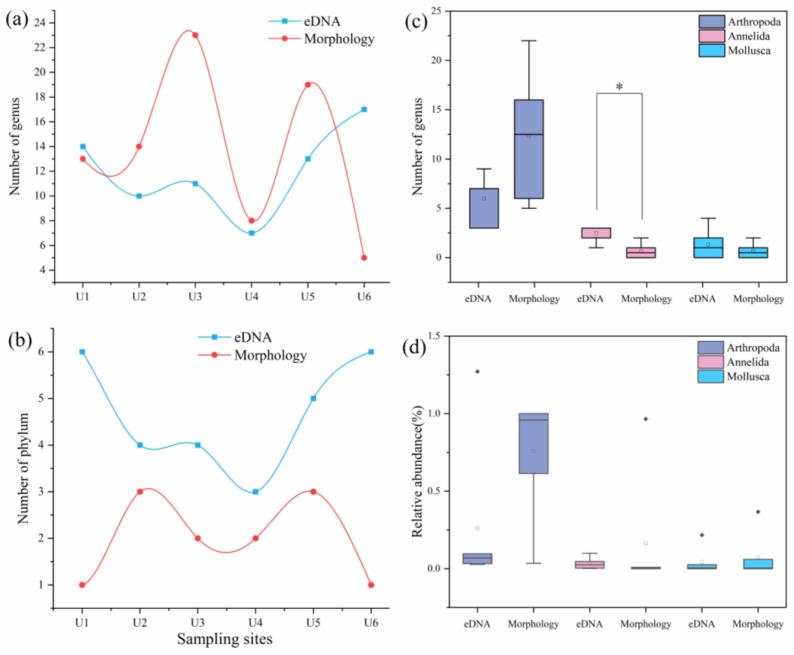
Benthic invertebrate richness at each site detected by eDNA metabarcoding and morphological methods at the genus (**a**) and phylum levels (**b**) and the number of genus-level taxa (**c**) and relative abundance (**d**) of shared phyla detected by both methods; * denote *p* ≤ 0.05.

The number of species of arthropods, annelids, mollusks, platyhelminthes, poriferans, and cnidarians detected by eDNA metabarcoding was 25, 9, 8, 2, 2, and 2, accounting for 52.08%, 18.75%, 16.67%, 4.17%, 4.17%, and 4.17%, respectively. Compared with traditional morphological methods, eDNA metabarcoding exhibited higher phylum-level detection at each site (Figure 2b). However, at the order level, the richness obtained with eDNA metabarcoding was greater than that obtained with morphology at sites U1, U2, U5, and U6; at the family level, the richness obtained with eDNA metabarcoding was greater than that obtained with morphology at sites U1, U2, and U6; at the genus level, the richness obtained with eDNA metabarcoding was greater than that obtained with morphology at only sites U1 and U6 (Figure 2a and Table A1). Among the benthic invertebrate communities detected by eDNA metabarcoding, that from site U4 had the lowest richness at all the taxonomic levels, which was consistent with the habitat conditions of site U4 observed during sampling. The Mann–Whitney *U* test revealed that the two methods exhibited significant differences in the richness of each locus only at the phylum level (Mann–Whitney *U* test: phylum: *Z* = −2.761, *p* = 0.006; order: *Z* = −1.149, *p* = 0.250; family: *Z* = −0.484, *p* = 0.629; genus: *Z* = −0.482, *p* = 0.630). In addition, the species richness of arthropods detected via eDNA metabarcoding was lower than that detected via morphological methods, whereas the species richness of mollusks detected via eDNA metabarcoding was greater than that detected via morphological methods, and the richness of annelid species detected via eDNA metabarcoding was significantly greater than that detected via morphological methods (Figure 2c and Table A2) (Mann–Whitney *U* test: Arthropoda: *Z* = −1.615, *p* = 0.106; Annelida: *Z* = −2.569, *p* = 0.010; Mollusca: *Z* = −0.763, *p* = 0.445). However, the largest difference in community species richness monitored by the two methods was in the phylum Arthropoda.

**Figure 3:**
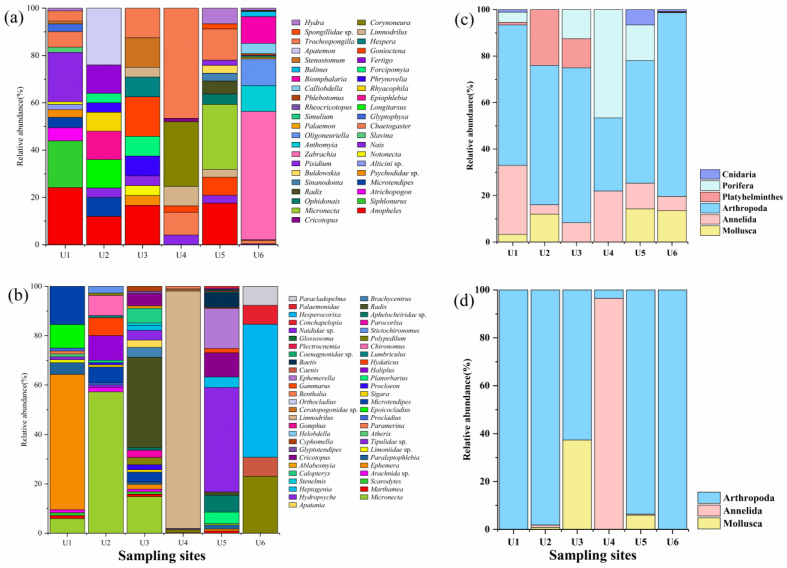
Relative abundance (%) of benthic invertebrates at the genus level (**a**,**b**) and phylum level (**c**,**d**) at each site using eDNA metabarcoding (**a**,**c**) or morphological methods (**b**,**d**).

In terms of the relative abundance of benthic invertebrates, at the genus level, the largest relative abundance measured on the basis of morphology was for *Limnodrilus* (16.04%), followed by *Micronecta* (13.01%) and *Ephemera* (9.64%). The genus with the highest relative abundance detected by eDNA metabarcoding was *Trochospongilla* (12.81%), followed by *Anopheles* (11.75%) and *Zabrachia* (9.04%). At the family level, Corixidae, detected on the basis of morphology, was the most dominant family (22.95%), whereas Spongillidae was the dominant family (13.18%) when detection was performed by eDNA metabarcoding. At the order level, Hemiptera was the most dominant order detected by the traditional morphological method (24.27%), whereas Diptera was the most dominant order detected by the eDNA metabarcoding method (33.99%). The relative abundances of arthropods, annelids, and mollusks determined via eDNA metabarcoding were not significantly different from those determined via the traditional morphological method (Mann–Whitney *U* test: Arthropoda: *Z* = −1.444, *p* = 0.149; Annelida: *Z* = −1.290, *p* = 0.197; Mollusca: *Z* = −0.000, *p* = 1.000) (Table A3), but compared with morphology, eDNA metabarcoding seemed to have a greater overall detection efficiency for Annelida (Figure 3c,d).

**Figure 4:**
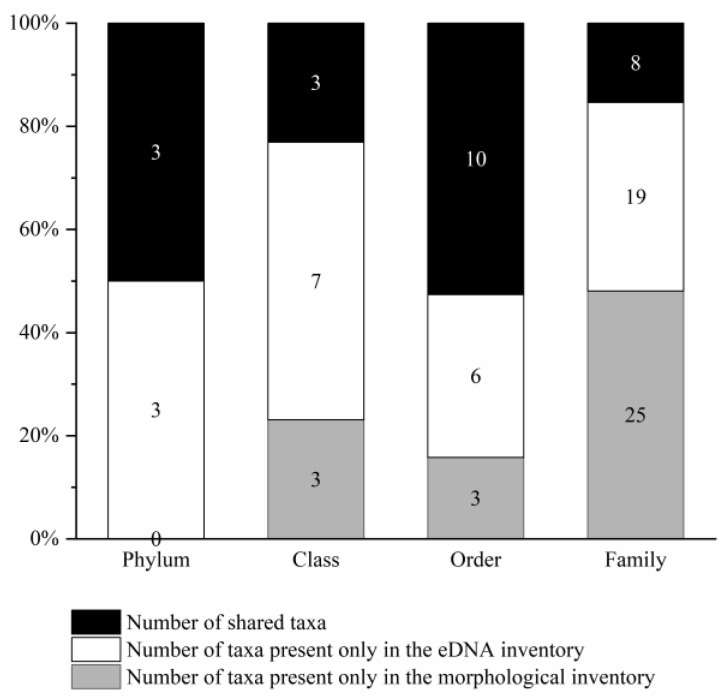
Number of taxa shared between the eDNA metabarcoding and morphology results at different taxonomic levels.

The number of shared units and each taxonomic level were compared between the results from morphology and eDNA metabarcoding; at the order level, eDNA metabarcoding showed the ability to detect more species that are small in size and are difficult to collect and identify by traditional morphological methods, including species belonging to Platyhelminthes, Porifera, and Cnidaria. At the class level, the shared taxa included Insecta, Oligochaeta, and Gastropoda, and eDNA metabarcoding detected a greater number of taxa. At the order level, six of the shared taxa were from Insecta. Among EPT species, eDNA metabarcoding detected only Ephemeroptera and Trichoptera species, and no Plecoptera species were detected. At the family level, the monitoring consistency of the two methods was low with only eight family-level taxa shared, and the morphological method revealed more taxa (Figure 4). At the genus level, the monitoring consistency of the two methods was even lower, with only five taxa shared. In summary, the consistency of using traditional morphological methods and eDNA metabarcoding to monitor benthic invertebrate communities in the Ulungu River Basin is low, but the complementarity is high.

### 3.2. Community Correlation Analysis

**Figure 5:**
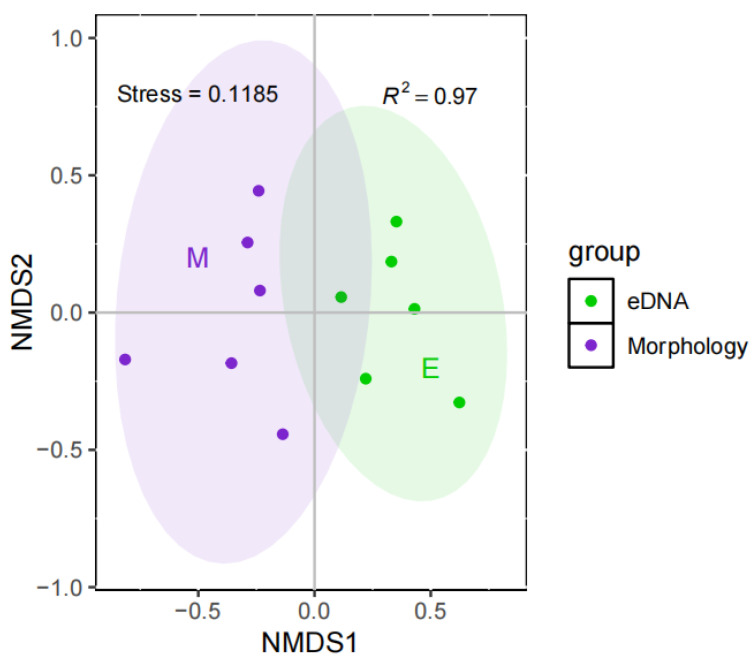
NMDS analysis of different benthic invertebrate communities monitored by eDNA metabarcoding and morphological methods.

The Mantel test was used to examine the correlations of different communities obtained via eDNA metabarcoding and traditional morphological methods, and the results revealed that the correlation between the two methods was not significant (*p* = 0.178). NMDS revealed extremely significant differences between different community collections obtained via the two methods (PERMANOVA, *p* = 0.0056; Figure 5), indicating that the monitoring consistency of the community structure via the two methods was low. When monitoring benthic invertebrate communities, the eDNA metabarcoding of water samples may still be unstable as an independent method.

### 3.3. Comparison of Ecological Status Assessments

**Figure 6:**
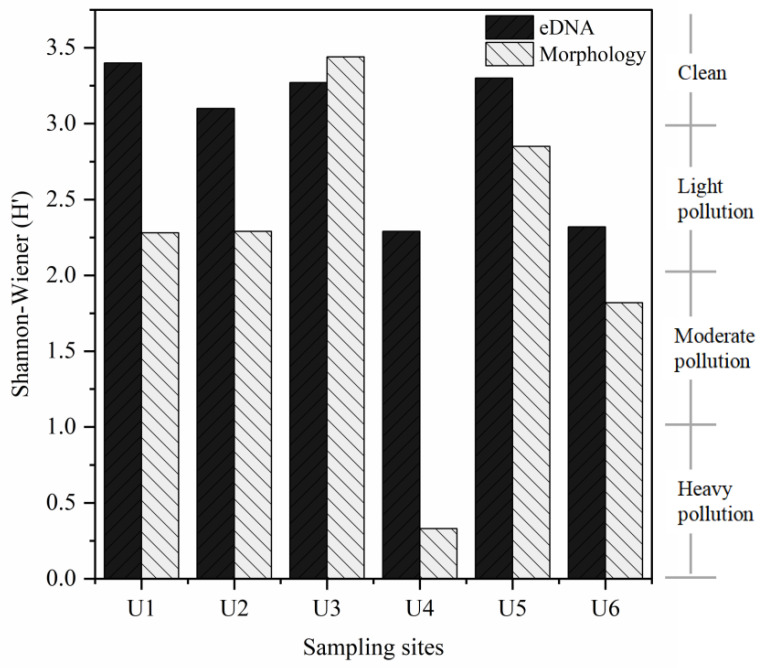
Shannon index and ecological status assessment at each site via eDNA metabarcoding and morphological methods.

The Shannon diversity index was calculated for each site via both methods. For eDNA metabarcoding, the H’ values of all the sites were above 2. The average value of H’ was 2.95, and the average ecological condition was light pollution. For the traditional morphological method, the average value of H’ was 2.17, the average ecological condition was light pollution, and the lowest value was 0.33 in U4 (Figure 6). The cor.test results showed that the correlation between the Shannon index at each site obtained via the two methods was not significant (*p* = 0.055). A comparison of the evaluation results of the two methods revealed that the eDNA metabarcoding and morphological methods revealed the same ecological assessment level at only the U3 site. In particular, at the U4 site, the morphological evaluation revealed heavy pollution, whereas the eDNA method revealed light pollution. However, according to the ecological conditions observed at the sampling site, the morphological assessment results were more consistent with the actual ecological conditions of site U4 at the time of sampling. A comprehensive comparison revealed that the average ecological grades of the two methods were consistent. However, when specific sites were compared, except for the U3 site, the ecological assessment results of eDNA metabarcoding were better than those of the traditional morphological method.

## 4. Discussion

At present, the method of obtaining eDNA from water samples to monitor aquatic ecosystems is widely used [38,39,40,41,42]. For example, Beermann et al. used water sample eDNA to investigate benthic animals in the Neijiang River in Chengdu, China, and obtained an abundance of species information [43]. This study compared the monitoring efficiency, similarity, and ecological assessment status of freshwater benthic invertebrate communities in the Ulungu River via water sample eDNA metabarcoding and traditional morphological methods. The consistency of the monitoring results between the two methods was low at the genus level. The number of shared taxa was small, and the community structure detected on the basis of eDNA in the water sample was extremely different from that determined by the morphological method (PERMANOVA, *p* = 0.0056). The differences in community structure are due to the following reasons: First, eDNA may integrate signals from multiple time points, while morphology only reflects community information at the moment of sampling, which may be one of the reasons for the differences; second, due to the limitations of the sampling method, morphology may miss some species, leading to differences in community composition, while eDNA may be affected by environmental disturbances such as hydrological pulses, leading to accelerated degradation; in addition, eDNA results are limited by database coverage, and primer preference may distort estimates of relative species abundance. In this study, the eDNA metabarcoding of water samples did not obtain more species information than morphology at a lower taxonomic level, which may prove the instability of eDNA in water samples. For example, environmental factors including high temperature, ultraviolet rays, and some microbial activities can accelerate the degradation of eDNA. In addition, hydrological disturbances such as excessive flow rate may dilute eDNA and resuspend historical signals. In addition, high turbidity can reduce filtration efficiency, and primer preference can lead to deviations.

In contrast, Ji et al. used sediment eDNA to study benthic invertebrates in urban rivers and found that they could detect significantly more species than morphology, and their detection efficiency of annelids was higher [44]. This is similar to the results of this study. The possible reason is that annelids are small in size and often live in the bottom mud, so traditional morphological sampling is easy to miss. Annelids have no exoskeleton on their bodies, and their metabolic secretions continuously release DNA. Their mitochondrial gene fragments are short and have strong resistance to degradation. eDNA can capture the DNA they release by filtering water samples. In addition, eDNA failed to detect more arthropods than morphology. The possible reason for this is that arthropods have complete exoskeletons, obvious morphological characteristics, slow DNA release, and are found with high accuracy in traditional classification. However, eDNA may be due to primer amplification bias. COI primers may have low binding efficiency for some groups (such as Pleuronectin in this study). In addition, arthropod DNA fragments are relatively long [45,46], which are more easily adsorbed and degraded by sediment. Rivera et al. used aquatic biofilms to collect eDNA and reported that the benthic community structure was similar to that determined via morphological methods [47]. More importantly, Múrria et al. reported that BULK sample DNA could be used to detect more abundant species than the morphological method and that eDNA metabarcoding and BULK samples may be used jointly to detect different and complementary parts of the community [48]. Lee et al. also showed that the communities determined from water sample eDNA differed from those determined on the basis of morphology and BULK samples [49], which is consistent with the low consistency between the two methods observed in our study.

In contrast to the results of the marine experiments, Antich et al. reported that even when eDNA was collected from water samples in the area closest to the benthic animal community, it was not able to accurately represent the structure of the benthic invertebrate community [50]. Willassen et al. also reported limited consistency between eDNA metabarcoding and morphological methods when studying the marine benthic invertebrates, but eDNA metabarcoding could detect additional or less obvious communities to supplement the results of morphological methods [51]. Therefore, the low consistency with the morphological results does not negate the monitoring value and cost-effectiveness of eDNA metabarcoding [52,53], but it cannot be denied that DNA collection methods that directly act on benthic invertebrates may be more efficient than eDNA-based methods. eDNA metabarcoding should improve the detection of rare samples and increase the sensitivity of most species in the environment [54]. This study emphasized that the current method of using water sample eDNA metabarcoding alone may reflect only part of the true benthic invertebrate community and may not be sufficient to reflect the most complete and true benthic invertebrate community, but this does not conflict with the view that water sample eDNA sampling may produce more reliable results.

eDNA metabarcoding detected benthic invertebrates belonging to 6 phyla, 10 classes, 16 orders, 27 families, 43 genera, and 48 species, whereas traditional morphological methods detected 3 phyla, 6 classes, 13 orders, 33 families, and 53 genera. eDNA metabarcoding can detect Platyhelminthes, Sponges, and Cnidaria, which are not identified by morphology. This may be due to the high sensitivity of eDNA to small and cryptic taxa, while traditional morphology is easily limited by sampling tools and identification difficulties, and DNA from non-biological sources may have limited contribution. At the order level and above, the eDNA metabarcoding method was used to monitor more abundant taxa, confirming that eDNA metabarcoding has greater monitoring efficiency at high taxonomic levels [55]. However, for EPT species, eDNA did not show good monitoring efficiency, and eDNA metabarcoding did not detect more taxa at levels below the family level. The low consistency between eDNA metabarcoding and morphological methods may be attributed to two factors: field sampling and laboratory methods [56]. First, in terms of sample collection, due to the long-term flow and replacement of water in rivers [57], the collected water sample eDNA may not accurately represent the true community species information of a certain site at a specific time. The species information obtained may also be detected by transporting or preserving the benthic invertebrates after death [58]. Moreover, compared with static water systems, flowing rivers can accelerate the degradation of eDNA [59]. In addition, there may be unavoidable exposure to the environment during the sampling process, and eDNA samples degrade faster than samples that are immediately returned or placed in a dark and frozen environment, even within 1 h [60,61]. Therefore, when using eDNA to monitor rivers, in future research, more environmental factors that control eDNA degradation need to be considered to optimize sampling strategies, such as biofilms, mineralogy, microorganisms, and chemicals [62,63]. This study has controlled for the possibility of eDNA degradation in water samples. Aseptic operation was performed during sampling, and light-shielded and low-temperature storage was performed during transportation to reduce the degradation effect. In addition, the water temperature was low during sampling (Table A4). Due to the dry season, the water flow rate was slow, and the sampling event was relatively reliable. However, environmental factors such as high sediment content, even at a slower flow rate, and salinity will undoubtedly still have an impact on eDNA. In addition, the selected primers may cause more inconsistent results between the two methods due to limitations such as preference. Although this study did not set up a negative control during the experiment, it has strictly controlled possible pollution and degradation links. It may have ignored the impact of the environment and experimental process on the results, but this procedural pollution may have limited interference with the results of this study.

In this study, classic COI universal primers that have been verified and are often used to monitor benthic invertebrates were used [33,34]. Ge et al. reported that COI gene fragments can significantly distinguish intra- and interspecies differences in benthic invertebrates [64]. Leese et al. reported that when universal primers were used to extract eDNA from water samples, many nontarget groups, such as plankton and fungi, were detected, resulting in the detection of benthic invertebrates and the dilution of OTUs. For this reason, they designed specific primers that could reduce the amplification of nontarget groups [65]. However, in terms of primer selection, few good choices exist at present, and some specific primers for benthic invertebrates have not been proven to be efficient and are therefore not widely used in other countries or regions worldwide. However, some studies have shown that by combining different types of markers, the data and ecological assessment results obtained are more consistent with the morphological results [66], which may provide new ideas and methods for eDNA metabarcoding. The eDNA extraction method may also be a factor affecting DNA yield and stability [67]. Therefore, it is necessary to standardize or optimize the key steps in the eDNA metabarcoding process to improve the stability of the method and the comparability of the results [68]. For example, in recent years, an invention patent has been registered for a self-sealing filter for a filtration device that collects eDNA from water samples. It uses synthetic resin materials to automatically dehydrate samples. The amount of DNA obtained is much greater than that obtained with ordinary filter membranes, and it can be used at room temperature. This filter does not degrade after being stored for several months. Another example is the optimization of water sample filtration strategies and the development of a high-capacity water sampling method to collect eDNA to improve detection sensitivity [69].

In this study, the ecological assessment results of eDNA metabarcoding and morphological methods were compared, and the results revealed that, except for the U3 site, the ecological assessment results of eDNA metabarcoding were better than those of traditional morphological methods, and the correlation between the ecological status results obtained by the two methods was not significant. eDNA may include historical signals (such as DNA from dead individuals) or cross-border DNA (such as the release of fish intestinal contents), leading to an overestimation of ecological status, while morphology reflects the immediate living community, and the two have different assessment dimensions. The water quality assessment results of the two methods at the U3 site are consistent. In the morphological method, the morphological diversity level of the U3 site is higher than that of the other sites, and in the eDNA method, the eDNA diversity level of the U3 site is similar to that of the other sites. Therefore, the “abnormality” of the lower Shannon index of eDNA compared to that of the morphological Shannon index at the U3 site may be caused by the limitations of the eDNA method, such as missed detections caused by primer preference. Therefore, in rivers in arid areas, eDNA ecological assessment needs to be combined with morphology to verify key groups (such as EPT insects) to avoid misjudgment by a single technology. Moreover, ecological assessment results are inevitably affected by the low consistency of detection results between eDNA metabarcoding and morphological methods. However, although eDNA metabarcoding can sometimes detect more abundant species than morphological methods, there is also the possibility that it cannot accurately provide reliable abundance estimates and subsequent ecological assessments [70]. However, before the water sample eDNA-based method is applied in ecological assessment, it should first be ensured that the target species in the environment can be accurately identified [71]. At present, the greatest challenge in the use of benthic invertebrate eDNA for ecological assessment may be the improvement of the index and the optimization of the barcode library [72], which should allow further development of scoring schemes for commonly used indices [73]. Since water sample eDNA and morphology may be used to monitor different or complementary parts of the community, the use of water sample eDNA for ecological assessment may also require new indicators [46]. Obtaining water sample eDNA data itself is challenging, and when conducting ecological monitoring, the indicators and methods used may not fully cover the scope and details of the data generated by eDNA metabarcoding. There are currently studies citing a new machine learning method model that can comprehensively use eDNA data. This combined method can replace morphological methods and may yield more accurate and realistic ecological assessment results [74,75]. In addition, there is currently little research on the DNA of benthic invertebrates in the Ulungu River. Therefore, in this study, a local eDNA database of benthic invertebrates in the Ulungu River was constructed to provide a basic reference for future research and the ecological protection of benthic invertebrates in the Ulungu River.

Ultimately, the eDNA metabarcoding method has long-term ecological research value and high cost-effectiveness. However, the key aspects of sampling, the control of the degradation rate, the eDNA extraction method, monitoring sensitivity, and data utilization still need to be considered. In addition, more research on and improvements in index development and other aspects should be conducted, with the goal of providing more convenient, more effective, safer, and more environmentally friendly methods for ecological research.

## 5. Conclusions

In this study, water sample eDNA-based methods and morphological methods were used to compare the community structure, diversity, and ecological assessment results of the benthic invertebrates in the Ulungu River Basin and initially construct a local benthic invertebrate eDNA database. eDNA metabarcoding did not detect more abundant species than morphological methods, and the two methods yielded fewer taxa at the family level and below. There were highly significant differences between the communities detected by the two methods (PERMANOVA, *p* = 0.0056). eDNA metabarcoding may reflect only part of the real benthic invertebrate community; however, it can be used to obtain the richness and abundance data of species that are difficult to identify morphologically, and these findings can be used to supplement morphological data. There was no significant correlation between the diversity and ecological assessment results of the two methods (cor.test, *p* = 0.055), and the ecological assessment results of eDNA metabarcoding cannot represent accurate and true ecological conditions. In summary, the water sample eDNA-based and the morphological methods have low consistency and high complementarity in monitoring benthic invertebrate communities and diversity, but the combination of the two methods can undoubtedly provide more precise and accurate ecological monitoring results. The protection of aquatic ecology on the basis of benthic invertebrates can reveal potential problems in an ecosystem in a timely manner, provide strong support for decision-making related to ecological protection, and have great significance for maintaining the balance of the ecological environment.

## Data Availability

Data will be made available on request.

## Appendix A

**Table A1:** The richness of benthic invertebrates at different taxonomic levels.

Sites		U1	U2	U3	U4	U5	U6
phylum	eDNA	6	4	4	3	5	6
	Morphology	1	3	2	2	3	1
order	eDNA	9	8	6	4	9	9
	Morphology	5	6	8	5	9	4
family	eDNA	12	10	10	5	10	14
	Morphology	9	8	15	6	18	4
genus	eDNA	14	10	11	7	13	17
	Morphology	13	14	23	8	19	5

**Table A2:** Genus-level richness of Arthropoda, Annelida, and Mollusca.

Sites		U1	U2	U3	U4	U5	U6
Arthropoda	eDNA	7	7	7	3	3	9
	Morphology	13	12	22	6	16	5
Annelida	eDNA	3	1	2	3	3	3
	Morphology	0	1	0	2	1	0
Mollusca	eDNA	1	1	0	0	4	2
	Morphology	0	1	1	0	2	0

**Table A3:** The relative abundances of Arthropoda, Annelida, and Mollusca.

Sites		U1	U2	U3	U4	U5	U6
Arthropoda	eDNA	0.6044	0.6	0.6667	0.3151	0.5275	0.7900
	Morphology	1	0.9818	0.6139	0.0349	0.9359	1
Annelida	eDNA	0.2967	0.04	0.0833	0.2192	0.1099	0.0617
	Morphology	0	0.0091	0	0.9651	0.0043	0
Mollusca	eDNA	0.0330	0.12	0	0	0.1429	0.1348
	Morphology	0	0.0091	0.3663	0	0.0598	0

**Table A4:** Water temperature at each site.

Parameters/Sites	U1	U2	U3	U4	U5	U6	Mean
Water Temperature/°C	7.9	11.4	11.3	12.6	14.4	14.1	12.0
